# Cross‐sectional study exploring the association between stressors and burnout in junior doctors during the COVID‐19 pandemic in the United Kingdom

**DOI:** 10.1002/1348-9585.12311

**Published:** 2022-01-13

**Authors:** Anli Yue Zhou, Mark Hann, Maria Panagioti, Mumtaz Patel, Raymond Agius, Martie Van Tongeren, Aneez Esmail, Peter Bower

**Affiliations:** ^1^ Division of Population Health, Health Services Research & Primary Care National Institute for Health Research School for Primary Care Research University of Manchester Manchester UK; ^2^ Division of Population Health, Health Services Research & Primary Care Centre for Occupational and Environmental Health University of Manchester Manchester UK; ^3^ Division of Population Health, Health Services Research & Primary Care Centre for Biostatistics University of Manchester Manchester UK; ^4^ Health Education England Manchester UK

**Keywords:** burnout, covid‐19, junior doctors, stressors, training

## Abstract

**Objectives:**

This study aims to develop a comprehensive list of stressors relevant to junior doctors and will also report findings exploring the associations between burnout and stressors, which include work and non‐work–related stressors as well as pandemic‐related stressors.

**Methods:**

An anonymous online questionnaire was sent to 1000 randomly selected junior doctors in the North‐West of England. The questionnaire included 37 questions on general and pandemic‐specific stressors, and the Maslach Burnout Inventory Health Services Survey. The main outcomes of interest were junior doctor ratings of stressors and scores for burnout (emotional exhaustion [EE], depersonalisation [DP], and personal accomplishment [PA]). Stepwise regression analysis was undertaken to assess associations between stressors and burnout.

**Results:**

In total, 326 responses were collected (response rate = 33%). Of the top 10 stressors rated by junior doctors, 60% were related to the pandemic. Multiple stressors were found to be associated with the burnout dimensions. Fatigue (*β* = .43), pandemic‐related workload increase (*β* = .33), and feeling isolated (*β* = .24) had the strongest associations with EE, whereas fatigue (*β* = .21), uncertainty around COVID‐19 information (*β* = .22) and doing unproductive tasks (*β* = .17) had the strongest associations with DP. Working beyond normal scope due to COVID‐19 (*β* = −.26), not confident in own ability (*β* = −.24) and not feeling valued (*β* = −.20) were found to have the strongest associations with PA.

**Conclusions:**

Junior doctors experience a combination of general stressors and additional stressors emerging from the pandemic which significantly impact burnout. Monitoring these stressors and targeting them as part of interventions could help mitigating burnout in junior doctors.

## 1 INTRODUCTION

Burnout is an occupational phenomenon caused by chronic workplace stress and the Maslach Burnout Inventory (MBI) has defined burnout into three dimensions: emotional exhaustion (EE), reduced sense of personal accomplishment (PA), and depersonalisation (DP).^1^ Doctors have been found to experience high levels of burnout worldwide^2^ and a recent study from the United Kingdom (UK) showed that nearly 1/3 doctors were experiencing signs of burnout.^3^ In particular, doctors earlier in their career were found to be at higher risk of burnout compared to more experienced doctors^4^ suggesting burnout may vary at different career stages.

Junior doctors working in the UK are qualified doctors engaging in formal postgraduate training and considering the high prevalence of burnout in junior doctors,^5^ it is important to explore which factors are driving stress and burnout in this group. Stressors are factors which may contribute to or precipitate stress and in our recent meta‐analysis, we found that organisational stressors such as work demands, poor work environment, and concerns about patient care had the strongest association with burnout compared to non‐work–related factors and non‐modifiable factors such as age and grade.^6^ However, there was wide variation in the stressors explored, assessment methods and in study quality which in turn may not enable definitive conclusions of which stressors are specific and relevant to junior doctors.^6^ Furthermore, current measures available in the literature may not comprehensively evaluate which particular stressors are most likely to encountered by junior doctors.^7^, ^8^, ^9^, ^10^, ^11^

The COVID‐19 pandemic has exacerbated the ongoing pressures experienced by doctors.^12^, ^13^ The pandemic in particular has impacted on junior doctors^14^ with a recent survey on over 28 000 UK junior doctors showing that 25% reported high levels of burnout, and 40% were experiencing EE during the COVID‐19 pandemic.^14^ This could be due to increased levels of existing stressors or pandemic‐specific stressors such as fear of transmission and concerns around personal protective equipment.^12^

Burnout has been found to be associated with the poorer quality of patient care and negative career outcomes for doctors,^15^ both of which may cause major pressures on the healthcare system. Hence, identifying underlying stressors that are associated with burnout in junior doctors is important for patients, doctors and the healthcare system as a whole. As current measures may not be comprehensive nor specific to the needs of this professional group, we aim to develop a comprehensive list of stressors relevant to junior doctors, which will include general stressors present before the pandemic as well as pandemic‐specific stressors, and to assess which of these stressors are most strongly associated with burnout.

## 2 METHODS

This was a cross‐sectional study using a self‐reported online questionnaire involving 1000 randomly selected junior doctors taking place between 10/07/20 to 04/08/20 in the North West region of England.

### 2.1 Study population

**FIGURE 1:**
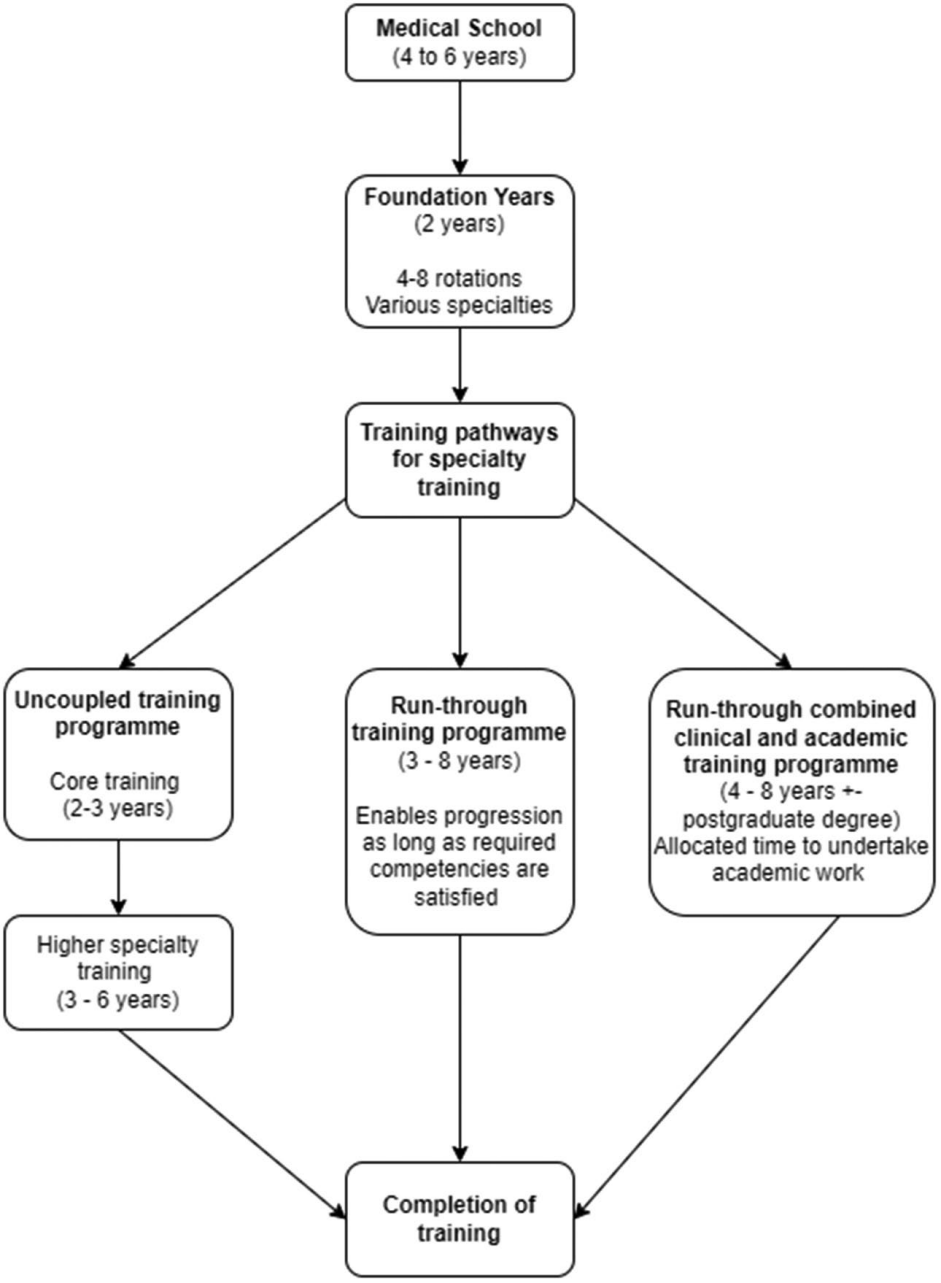
Training pathways for junior doctors working in the UK

In the UK, all junior doctors are employed in standardised training posts and follow set training pathways within a region to enable them to become accredited specialists within their chosen specialty (Figure 1).

After graduation, all junior doctors undertake a 2‐year Foundation training rotation that provides experience in various specialties. Following this, junior doctors will join a specialty pathway which usually involves core training (2–3 years) followed by more specialised training (3–6 years). General Practice trainees will engage in a 3‐year programme after completing their Foundation training, whereas specialty training can take more than 8 years.

The population of interest in this study included junior doctors who were currently engaging in postgraduate training in the North West region of England (*n* = 7121).

### 2.2 Study measures

The questionnaire consisted of 66 questions covering 7 demographic questions, general and COVID‐19 related stressors experienced over the past month (37 questions using a 7‐point Likert scale), and MBI Health Services Survey (22 questions).

### 2.3 Demographic characteristics

The following items were collected: sex, age interval, specialty, grade, full‐time status, ethnicity, and place of qualification. Age intervals were used instead of actual age due to confidentiality concerns. An option of “prefer not to answer” was also available.

### 2.4 General stressors

The general stressors questionnaire consisted of 25 questions compiled from a qualitative study on junior doctors in the same region, and a systematic review and meta‐analysis that focused on stressors in junior doctors.^6^, ^16^ A 7‐point Likert scale was used as this has been found to have higher reliability compared to 5‐point Likert scales.^17^ Participants were asked to choose an option between “strongly disagree” (score = 0) to “strongly agree” (score = 6) for each stressor over the past month. The option of “not applicable” was also included as we appreciate not all stressors may be relevant to some groups of junior doctors, for example, on‐call commitments.

Out of the 25 questions, seven of these questions were related to non‐work–related stressors whereas the other 18 questions were related to work‐related stressors.

An external pilot study on 20 junior doctors was undertaken in August 2019 to receive feedback on the questionnaire content and format.

### 2.5 COVID‐19 stressors

The COVID‐19 questionnaire consisted of 12 questions, of which 6 were related to personal stressors and the other 6 were related to organisational stressors. A 7‐point Likert scale was used and participants were asked to choose an option between “strongly disagree” (score = 0) to “strongly agree” (score = 6) for each stressor over the past month, and a “not applicable” option was also included in the questionnaire for each stressor.

Due to the impact of the COVID‐19 pandemic on mental health in doctors^12^ and the time critical nature of trying to capture data on stressors, a scoping literature search was undertaken in June 2020. The search strategy used in Zhou et al.^6^ was used with the additional terms (Covid‐19 OR coronavirus OR severe acute respiratory syndrome OR SARS OR Middle East respiratory syndrome OR MERS OR pandemic). The review identified 21 pandemic‐related stressors. In order to identify the most relevant pandemic related stressors, we approached relevant stakeholders and experts (junior doctor wellbeing researchers, British Medical Association (BMA) Junior Doctor Committee, Royal College of Physicians representatives, Health Education England (HEE) and Occupational Health). Stakeholders and experts were asked to rank the 10 most relevant stressors in the list and provide feedback on any pandemic‐related stressors which they felt were missing from the list. Following this, the number of recommendations by the experts/stakeholders were added together with the number instances the stressor had been mentioned within the scoping literature review to create a total of 12 relevant COVID‐19 items which did not overlap with the 25 general stressor questions.

### 2.6 Burnout

Burnout in junior doctors was measured using the MBI Health Services Survey, which is a validated 22‐item questionnaire (using a 7‐point Likert scale) and is the gold standard for measuring burnout.^1^, ^4^ The questionnaire contains three subscales and measures the three individual domains of burnout: EE, DP, and PA.^1^ The MBI has been shown to have good internal consistency (EE = 0.90, DP = 0.79, PA = 0.71) and has also shown to have good convergent and divergent validity.^1^

### 2.7 Study procedure

A pre‐warning email to inform participants about the upcoming questionnaire was sent on 07/07/20 to the 1000 junior doctors and the email also contained a participant information sheet. The formal invitation containing the questionnaire link was sent to participants via email by HEE on 10/07/20. Non‐respondents received two additional reminders on 20/07/20 and 29/07/20 by email through HEE. The survey was live between 10/07/20 to 04/08/20.

Previous studies have found that unconditional incentives rather than prize draws can improve response rates^18^ and all junior doctors who completed the questionnaire were given a £20 shopping voucher for their participation. Data collection was conducted through Sawtooth Survey Software.^19^

### 2.8 Sample selection

A random sample of 1000 junior doctors were selected from the eligible population of 7121 using STATA 14.0.^20^ This sample was found to be representative of the junior doctor population in terms of age, gender, specialty, grade, and country of qualification.

Surveys involving doctors tend to have lower response rates than the general population^21^ and it is not uncommon to see response rates of less than 40%.^21^, ^22^, ^23^, ^24^ Assuming we will get 300 responses (30% response rate^21^, ^22^, ^23^, ^24^), the study can estimate the associations between stress items and sub‐scales of the MBI as small as 0.20 with in excess of 90% power.

### 2.9 Data analysis

To identify which stressors were felt to be more important by junior doctors, we calculated percentages of “moderately agree”/”strongly agree” responses, mean and median ratings (based on the Likert‐scale) and then ranked the stressors.

Previous literature suggests that the MBI dimensions are better handled as continuous variables.^25^ Considering forward stepwise regression may be associated with a suppressor effect and may not detect important covariates,^26^ backwards stepwise regression was undertaken to identify which stressors were associated with the burnout dimensions (EE, DP, and PA). All demographic characteristics such as age interval, full‐time status, gender, ethnicity, country of graduation and grade were controlled for in the stepwise regression analysis. The least significant stressors were sequentially removed from the model until all the remaining items were significant at the nominal 5% level. A forwards stepwise regression model was also undertaken which confirmed the stepwise regression results were consistent and did not identify any additional stressors. A two‐sided *P* ≤ .05 was considered statistically significant. Bootstrapping was undertaken to estimate model standard errors as it makes minimal assumptions about the distribution of the observed data.

Stressors that were found to have a statistically significant association with burnout dimensions underwent further analysis and beta coefficients were also reported in order to determine the relative strength of association of each stressor with the burnout dimensions. For EE and DP, a stronger positive association is suggestive of a worse outcome whereas with PA, a stronger negative association is suggestive of a worse outcome. Data analysis was undertaken using STATA, version 14.0 (StataCorp).^20^

## 3 RESULTS

### 3.1 Demographics

**FIGURE 2:**
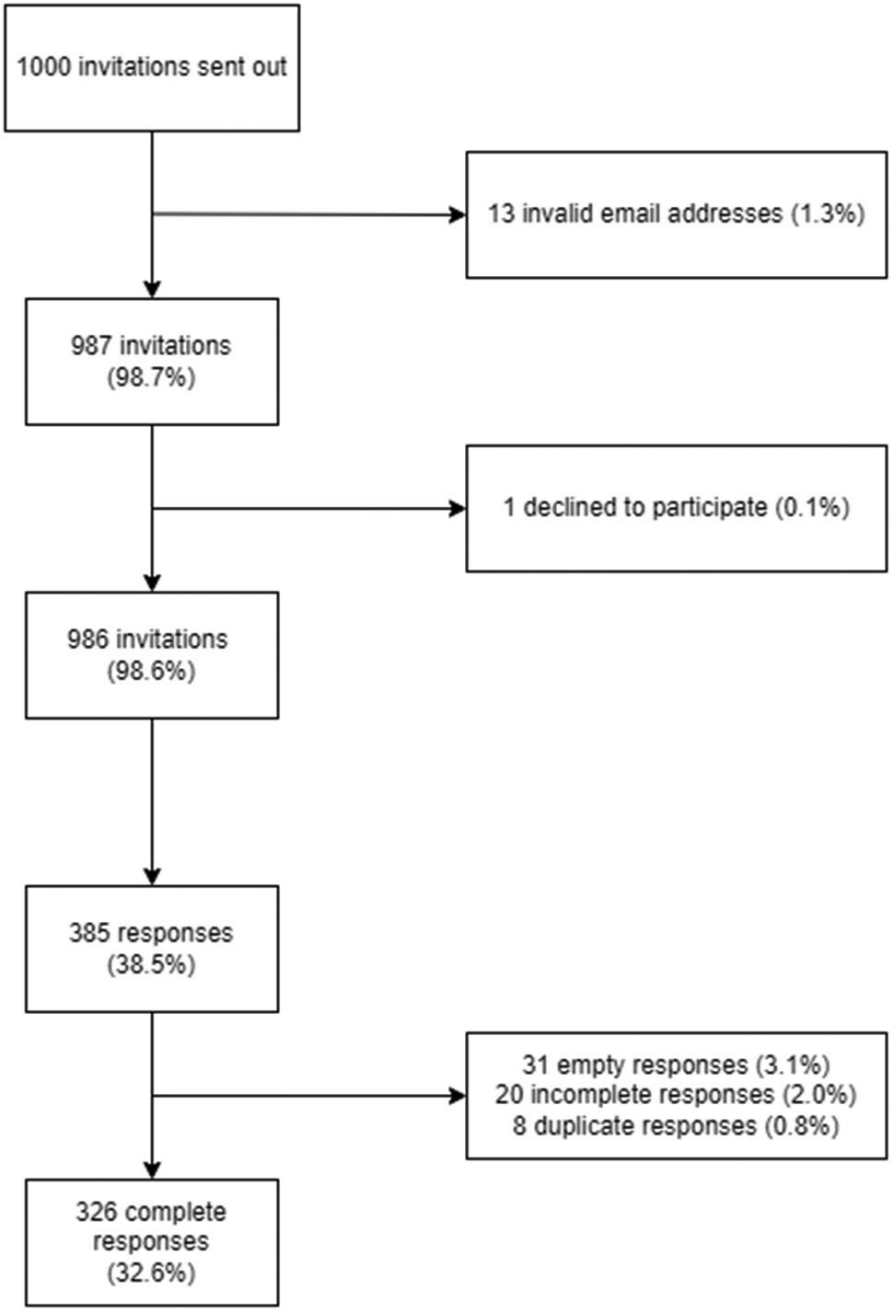
Flow process summarising the number of responses

A total of 326 complete responses were collected during the study period (Figure 2) giving a response rate of 33%.

Demographic results are presented in Table 1. Study respondents were representative of the North‐West junior doctor population in terms of gender and country of qualification. Age interval was collected in this study and therefore it was not possible to compare this with the junior doctor population. Ethnicity and full‐time status were not available to us in the junior doctor population dataset. In specialty, Foundation doctors who undertake rotation in various specialties were found to be under‐represented compared to other specialties (23.4% [junior doctor population] vs. 8.6% [current sample]), whereas it was noted that foundation grade doctors (in 1st or 2nd postgraduate year of training) were over‐represented in grade (23.4% [junior doctor population] vs. 30.1% [study sample]). However, when weighting was applied according to specialty and grade, the conclusions did not change when using a weighted analysis.

**TABLE 1:** Demographic results

Demographics (*n* = 326)	Frequency (%)	North‐West junior doctor population (*n* = 7121)
Age		
<30	198 (60.7)	—
31–35	89 (27.3)	—
>36	39 (12.0)	—
Gender		
Female	177 (54.3)	3794 (53.3%)
Male	149 (45.7)	3149 (44.2%)
Grade		
Foundation doctor	98 (30.1)	1666 (23.4%)
Core training‐ core training 1 or 2	96 (29.4)	2206 (31.0%)
Specialty training 3/core training 3	60 (18.4)	1100 (15.4%)
Specialty training 4/5	39 (12.0)	1158 (16.3%)
Specialty training 6+	33 (10.1)	991 (13.9%)
Specialty		
Anaesthetics	30 (9.2)	540 (7.6%)
Foundation	28 (8.6)	1666 (23.4%)
Other	47 (14.4)	1109 (15.6%)
Emergency medicine	21 (6.4)	243 (3.4%)
General practice	78 (23.9)	1576 (22.1%)
Medicine	66 (20.2)	890 (12.5%)
Surgery	38 (11.7)	687 (9.7%)
Paediatrics	18 (5.5)	410 (5.8%)
Full‐time status		
Full‐time	292 (89.6)	—
Less than full‐time	34 (10.4)	—
Ethnicity^a^		
White	174 (53.4)	—
Non‐white	150 (46.6)	—
Place of graduation		
United Kingdom	251 (77.0)	5550 (77.9%)
European economic area	9 (2.8)	258 (3.6%)
International medical graduate	66 (20.2)	993 (13.9%)^b^

^a^ 2 preferred not to disclose and therefore not included.

^b^ 4.5% (*n* = 320) were categorised as unknown place of graduation.

### 3.2 Burnout scores

Mean scores for EE, DP and PA were 25.2, 9.8 and 34.5, respectively, with 38%, 42% and 40% of the scores meeting the high burnout criteria for EE, DP, and PA, respectively (Table 2).

**TABLE 2:** Burnout scores

Descriptives	Emotional exhaustion	Depersonalisation	Personal accomplishment
Mean	25.2	9.8	34.5
Standard deviation	10.2	6.41	6.81
Median	24	9	36
Range	1–54	0–29	7–48
Skewness	0.31	0.52	−0.71
% of junior doctors reporting high scores based on published cut‐offs^4^	38	42	40

### 3.3 Stressors

The 37 stressors have been presented according to the frequency and impact of agree responses, median and mean scores in Table 3.

**TABLE 3:** Stressors which have been ranked according to the proportion of agree responses, mean and median

Stressor	% Moderately and strongly agree	Median	Mean
1. Duration of COVID‐19 pandemic^a^	70.3	5	4.73
2. COVID‐19 disrupting work‐life balance	64.4	5	4.98
3. Concerns about non‐COVID‐19 patient care	59.8	5	4.58
4. No control over frequent changes due to COVID‐19^a^	59.2	5	4.71
5. Fear of making mistakes	47.5	4	4.15
6. On call/out of hours work	47.2	5	4.05
7. Career development and progression	46.9	4	4.01
8. Passing on COVID‐19^a^	46.9	4	4.06
9. Training disrupted by COVID‐19	45.7	4	3.53
10. Unhealthy lifestyle^a^	42.6	4	3.83
11. Blame myself when things go wrong^a^	41.4	4	3.89
12. Doing unproductive tasks	39.6	4	3.88
13. High workload	39.3	4	3.74
14. Personal protective equipment concerns	36.5	4	3.97
15. Grief from COVID‐19 related deaths^a^	35.9	4	3.53
16. Feeling isolated due to COVID‐19^a^	35.9	4	3.93
17. Fatigue^a^	35.3	4	3.79
18. Training needs are not met	35	4	3.45
19. Cannot plan annual or study leave	31.6	4	3.42
20. Complaints or being under investigation	30.7	4	3.49
21. Poor worklife balance	29.1	4	3.35
22. Work commute^a^	25.2	3	2.92
23. Media reports negatively on junior doctors^a^	24.2	3	3.03
24. No control over work	23.6	3	3.19
25. Increase in workload and hours due to COVID‐19	22.7	3	2.9
26. Uncertainty around COVID‐19 information^a^	18.4	3	2.77
27. Working beyond normal scope due to COVID‐19	17.2	2	2.63
28. Not feeling valued	16.3	2	2.34
29. Personal circumstances (e.g. childcare, bereavement)^a^	16	3	2.53
30. Cannot take breaks	14.4	2	2.18
31. Negative work environment culture	14.1	2	2.28
32. Interpersonal difficulties	12	1	2.02
33. Lacking in my own abilities^a^	8.6	2	2.09
34. Cannot raise work concerns	8	1	1.86
35. Cannot take sick leave	7.4	1	1.42
36. No senior support	7.4	1	1.75
37. Not supported by colleagues	4.6	1	1.44

^a^ Non‐work–related stressors.

The four stressors with the highest proportion of “moderately” and “strongly agree” responses were related to COVID‐19, as were 60% of the top 10. In relation to the general stressors, the stressors with the highest proportion of “moderately” and “strongly agree” responses were related to “Career development and progression”, “Fear of repercussions from mistakes”, and “On‐call/out of hours work.” This remained the same when median and mean scores were compared. Non‐work–related factors related to COVID‐19 and “Unhealthy lifestyle” were also identified in the top 10 stressors.

The bottom four stressors with the lowest proportion of “moderately” and “strongly agree” responses were related to the work environment. All 10 stressors with the lowest proportion of “moderately” and “strongly agree” responses were related to non‐work or work‐related stressors rather than COVID‐19 related stressors.

### 3.4 Backwards stepwise regression results

General stressors and COVID‐19 stressors which were significant at the 5% level (*P* < .05) are presented in Table 4.

**TABLE 4:** General stressors and COVID‐19 stressors which are significant at the 5% level using backwards stepwise regression modelling

	Emotional exhaustion		Depersonalisation		Personal accomplishment	
	Coefficient (95% CI^a^)	*β*	Coefficient (95% CI^a^)	*β*	Coefficient (95% CI^a^)	*β*
General stressors						
Negative culture	1.00 (0.48, 1.58)	.18	—	—	—	—
Fatigue	2.74 (2.12, 3.34)	.43	0.91 (0.50, 1.30)	.21	—	—
Fear of making mistakes	0.79 (0.25, 1.42)	.19	—	—	—	—
Career development and progression	0.90 (0.36, 1.43)	.13	—	—	—	—
Unhealthy lifestyle	0.91 (0.34, 1.34)	.10	—	—	—	—
Doing unproductive tasks	—	—	0.57 (0.17, 0.94)	.17	—	—
Not confident in own abilities	—	—	—	—	−0.85 (−1.32, −0.37)	−.24
Not feeling valued	—	—	—	—	−0.63 (−1.02, −0.24)	−.20
No control over work	—	—	—	—	−0.53 (−0.95, −0.08)	−.09
COVID‐19 related stressors						
No control over frequent changes due to COVID‐19	1.13 (0.43, 1.96)	.17	—	—	—	—
Training disrupted by COVID‐19	−0.69 (−1.38, −0.08)	−.14	—	—	—	—
Feeling isolated due to COVID‐19	1.27 (0.68, 1.94)	.24	—	—	−0.58 (−1.02, −0.14)	−.17
Working beyond normal scope due to COVID‐19	0.97 (0.30, 1.52)	.14	—	—	−0.85 (−1.35, −0.42)	−.26
Increase in workload and hours due to COVID‐19	1.81 (1.13, 2.40)	.33	—	—	—	—
Uncertainty around COVID‐19 information	—	—	0.87 (0.34, 1.34)	.22	—	—
Passing on COVID‐19	—	—	—	—	0.52 (0.01, 1.00)	.13

^a^ Using bootstrapped percentile confidence intervals.

Most stressors were found to have a positive relationship with EE and DP, however “Training disrupted by COVID‐19” was found to have an inverse relationship to EE. Most stressors were found to have an inverse relationship with PA, except for “Passing on COVID‐19”. “Fatigue” was found to have a positive association with EE and DP. Both “Working beyond normal scope due to COVID‐19” and “Feeling isolated due to COVID‐19” was found to have a positive association with EE and negative association with PA.

## 4 DISCUSSION

### 4.1 Statement of principle findings

This regional cross‐sectional study has identified a comprehensive range of general work‐related, non‐work–related and COVID‐19 related stressors. Stressors across all subcategories were found to be associated with dimensions of burnout, especially EE. Although the three most frequently reported stressors in our study were not found to be associated with burnout, however the stressors with the highest impact in relation to frequency of reporting and associations with MBI dimensions were “Fear of making mistakes”, “No control over frequent changes due to COVID‐19”, “Career development and progression”, “Unhealthy lifestyle” and “Doing unproductive tasks”.

### 4.2 Strengths and weaknesses of the study

This study has identified a range of generic and pandemic‐specific stressors experienced by junior doctors working in the UK which could lead to burnout. This is also one of the very few studies that have focused specifically on junior doctors during the COVID‐19 pandemic.^27^, ^28^, ^29^ While stressors explored in these previous studies have been limited^28^, ^29^ our study has included stressors identified by UK junior doctors and the literature.^6^, ^16^ This study therefore provides important results to not only general stressors, but also COVID‐19 related stressors, which can guide intervention development to mitigate burnout in junior doctors. In the future, it may be beneficial to consider larger longitudinal studies covering the UK, to identify and monitor trends in stressors in the short and long term and potentially provide additional insights into which stressors are important predictors of burnout in junior doctors with the ultimate aim to address junior doctor burnout.

This was a cross‐sectional study and therefore causation cannot be assessed within this study. Our study had an overall response rate of 32.6%, which is reflective of previous online survey studies involving doctors.^22^, ^23^, ^24^, ^30^ Although our study attempted to optimise response rates through adopting various methods such as a covering letter, advanced warning email, giving a token of appreciation and having two follow up reminders,^17^, ^21^ our response rates remained similar to other studies.^22^, ^23^, ^24^, ^29^, ^30^ Survey fatigue is also an ongoing issue with a previous study predicting that over a quarter of junior doctors were experiencing survey fatigue.^31^ Considering our findings that COVID‐19 has affected junior doctors’ work life and training, with relatively high levels of burnout, it is likely that survey fatigue may have contributed to our response rate of 32.6%. Response bias may also have been present within our study as foundation doctors were found to be overrepresented compared to other grades. Grade was also not found to be associated with stress/burnout in a previous meta‐analysis^6^ and furthermore, we did control for demographics within our regression analysis. The authors did not have access to the junior doctor database due to data protection constraints therefore it was not possible to assess whether there were actual differences between participants and non‐participants, which may have helped us understand whether there were any demographic factors contributing to response bias within our study. Our results also focused in the North West region of England and therefore may not be representative of the UK junior doctor population or to junior doctors in other countries, therefore may also benefit from UK‐wide and international longitudinal studies.

### 4.3 Comparison with other studies

Frequently reported work‐related factors such as concerns about career development, fear of making mistakes and unproductive tasks were found to be significantly associated with burnout dimensions in our study which is consistent with previous literature.^15^ These stressors are likely to have been perpetuated by the ongoing COVID‐19 pandemic^32^ and junior doctors caring for COVID‐19 patients have been found to have higher levels of burnout compared to those who were not exposed.^29^ This suggests that although additional stressors may have emerged from the pandemic, pre‐existing stressors may continue to be associated with burnout in junior doctors.

The General Medical Council (GMC) National Training Survey is a mandatory survey in which junior doctors are asked about their training in order to monitor the quality of training in the UK.^14^ The survey explores specific training factors and also includes components of the Copenhagen Burnout Inventory (CBI).^14^ Their results confirmed that over 40% of junior doctors reported heavier workloads and 74% reported training disruptions during the pandemic. However, questions related to training were not explored in the context of stress and burnout and associations between training factors and the CBI were not assessed, therefore it was not possible to identify predictors or drivers of burnout within this survey. Furthermore, the GMC National Training Survey only includes work‐related burnout components whereas Kristensen et al. recommends including personal burnout to differentiate workers who may be experiencing burnout due to non‐work–related factors.^33^ Kristensen et al. argues that the MBI general survey is too generic for workers involved in human services related work,^33^ however there was no discussion or comparison with the MBI human services survey which was used in our study and this may be an area that warrants further research. In comparison to the GMC National Training Survey, our study specifically explored the association between stressors and validated burnout dimensions, which not only identified workload and training disruptions as important stressors, but also identified associations with burnout dimensions. However, both our study and the GMC National Training Survey did not specifically assess whether the perceived workload was related to longer working hours or pacing intensity of work tasks, which could be an area of further research, as it would provide additional understanding to how workload is perceived by junior doctors.

Unhealthy lifestyle factors have been found to be associated with occupational stress and burnout in doctors^6^, ^34^, ^35^, ^36^ and our results showed that unhealthy lifestyle factors are associated with burnout. Burnout has been associated with fast food consumption and reduced exercise in doctors,^34^ and have been found to feel more stressed after engaging in unhealthy eating patterns such as binge eating.^35^ Factors such as redeployment to COVID‐19 frontline wards, changes to rotations and shift patterns at short notice, and working longer hours due to staff shortages^37^ could have contributed to this as junior doctors may prioritise patient care and work over their own health. Unhealthy lifestyle factors were found to be an important stressor by junior doctors in our study, and this may be an area where interventions could be focussed on in future research.

An interesting result from our study was that relatively few junior doctors did report a lack of support in their daily work life, but those who did had a higher risk of EE. A recent survey on morale of redeployed junior doctors during the pandemic supports this finding with junior doctors indicating that they feel valued by their team.^37^ Similar results were also reflected in the 2020 GMC National Training Survey, which showed that over 80% of junior doctors surveyed felt there was a culture of teamwork and a supportive environment.^33^ Peer and senior support has previously been found to help junior doctors cope with stress.^16^ Although COVID‐19 has increased work demands on doctors, it is apparent that practical support measures made during the COVID‐19 pandemic such as peer support, rest facilities as well as receptiveness to staff feedback can minimise the negative impact of burnout in junior doctors.^27^, ^29^

### 4.4 Meaning of the study: Possible explanations and implications for clinicians and policymakers

Our results suggest that although training and workload are important stressors, however, junior doctors have also been concerned about their health and issues arising from the COVID‐19 pandemic such as feelings of isolation and lack of control. Junior doctors may engage with social distancing to reduce the transmission risk of COVID‐19 to family and friends, which in turn may remove the social contact and support they may usually receive,^16^ and can lead to isolation and uncertainty. Furthermore, with increasing workloads and additional demands in the workplace as identified in our study, junior doctors may not have the time or the motivation to engage with healthy lifestyle measures, which are usually utilised as coping mechanisms to help mitigate stress and burnout.^16^ Considering poor perceived health has been associated with burnout^6^ and the fact that only 66% of junior doctors rated workplace wellbeing support as good/very good,^14^ stakeholders may wish to consider targeting resources to improve workplace wellbeing and healthy lifestyles in junior doctors, which in turn may mitigate burnout.

Our study also found fatigue to be an important stressor associated with burnout, especially EE and DP, and similar findings have been previously found in other doctors in training.^38^ EE has been found to be related to feelings of fatigue,^39^ but may also be a consequence of chronic sleep deprivation and managing conflicting work and training demands, with demands in their personal life. Fatigue has been associated with negative outcomes such as medical errors^38^ and therefore it is important to identify suitable interventions to mitigate fatigue in the workplace. Previous national initiatives have emphasized the importance of fatigue, but this has not always led to practical changes in the workplace.^40^ National funding was provided in 2019 to fund the BMA Fatigue and Facilities charter which sets standards to improve work facilities and minimise fatigue in the workplace for junior doctors, however it is unclear how the charter is being implemented by different employers and how effective the measures are in improving fatigue in junior doctors.^32^ Fatigue Risk Management Systems have been introduced as a systematic method to monitor fatigue in the workforce and to identify areas which may be hazardous with the aim to develop measures to mitigate risk; however, this practice is not routinely implemented in the healthcare sector^40^ and could be an area for further exploration and development.

Both work and non‐work–related stressors were found to be associated with burnout suggesting burnout is multifactorial; therefore, targeting one aspect may not be sufficient to address burnout. Our findings reinforces that burnout can stem from organisational issues, but non‐work–related stressors can also be exacerbated by work and therefore it may benefit from multilevel interventions targeting a range of different stressors^15^ as identified in our study such as training and career development, work environment and demands, uncertainty and risk, as well as personal factors. Using a toolkit of wellbeing initiatives alongside organisational interventions may help mitigate negative outcomes such as burnout. Moreover, as modern clinical practice develops overtime, the stressors affecting junior doctors may also change and therefore there is a need to develop a tool which can identify and monitor stressors^13^ that are important to junior doctors in order to develop and adapt interventions that are effective in mitigating the risk of negative outcomes such as burnout.

## 5 CONCLUSIONS

Our study has identified that multiple general and COVID‐19 specific stressors in junior doctors which contribute to burnout. These included both work and non‐work stressors further supporting the need for multilevel interventions targeting work environment and work demands as well non‐work–related factors that could be exacerbated by workplace stressors such as fatigue and unhealthy lifestyles. Finally, our findings highlight the importance of developing a tool that can measure, monitor and identify important stressors in junior doctors with the aim for this to be integrated into risk management systems to guide future intervention development.

## DISCLOSURE

*Approval of the research protocol*: Approval was received from the University of Manchester Research Ethics Committee (UREC5‐2016‐0093‐462) and the research committee at HEE North‐West. *Informed consent*: Consent was implied by survey completion. *Registry and the registration no. of the study/trial*: Approval was received from the University of Manchester Research Ethics Committee (UREC5‐2016‐0093‐462) and the research committee at HEE North‐West. *Animal studies*: N/A. *Conflict of interest*: The authors declare no conflict of interest for this article.

## AUTHOR CONTRIBUTIONS

Anli Yue Zhou designed the study, designed data collection tools, monitored data collection, cleaned, analysed and interpreted the data, and drafted, revised and approved the paper. Mark Hann contributed to study design, monitored data collection, contributed to data analysis and interpretation, and contributed to, revised critically and approved the paper. Maria Panagioti contributed to study design, monitored data collection, data interpretation, and contributed to, revised critically and approved the paper. Mumtaz Patel contributed to study design and data collection, and revised critically and approved the paper. Raymond Agius contributed to study design, and revised critically and approved the paper. Martie Van Tongeren and Aneez Esmail both contributed to data interpretation, and revised the paper critically and approved the paper. Peter Bower contributed to study design, monitored data collection, data interpretation, and contributed to, revised critically and approved the paper.

## Data Availability

The data that support the findings of this study are available from the corresponding author upon reasonable request.
